# Supplementary material for: Upper Critical Solution Temperature (UCST) Behavior of Polystyrene-Based Polyampholytes in Aqueous Solution
Source: Polymers (Basel). 2019 Feb 4;11(2):265. doi: 10.3390/polym11020265 (PMC6419060; doi:10.3390/polym11020265)
Supplement: Supplementary file 1 [file polymers-11-00265-s001.pdf]

Supporting Information

Upper Critical Solution Temperature (UCST) Behavior of Polystyrene-based Polyampholytes in Aqueous Solution

Komol Kanta Sharker[1], Yuki Ohara[1], Yusuke Shigeta[2], Shinji Ozoe[2] and Shin-ichi Yusa[1,*]

[1] Department of Applied Chemistry, Graduate School of Engineering, University of Hyogo, 2167 Shosha, Himeji, Hyogo 671-2280, Japan

[2] Tosoh Finechem Co., 4988 Kaisei-cho, Shunan, Yamaguchi 746-0006, Japan

* Correspondence: yusa@eng.u-hyogo.ac.jp; Tel: +81-79-267-4954; Fax: +81-79-266-8868

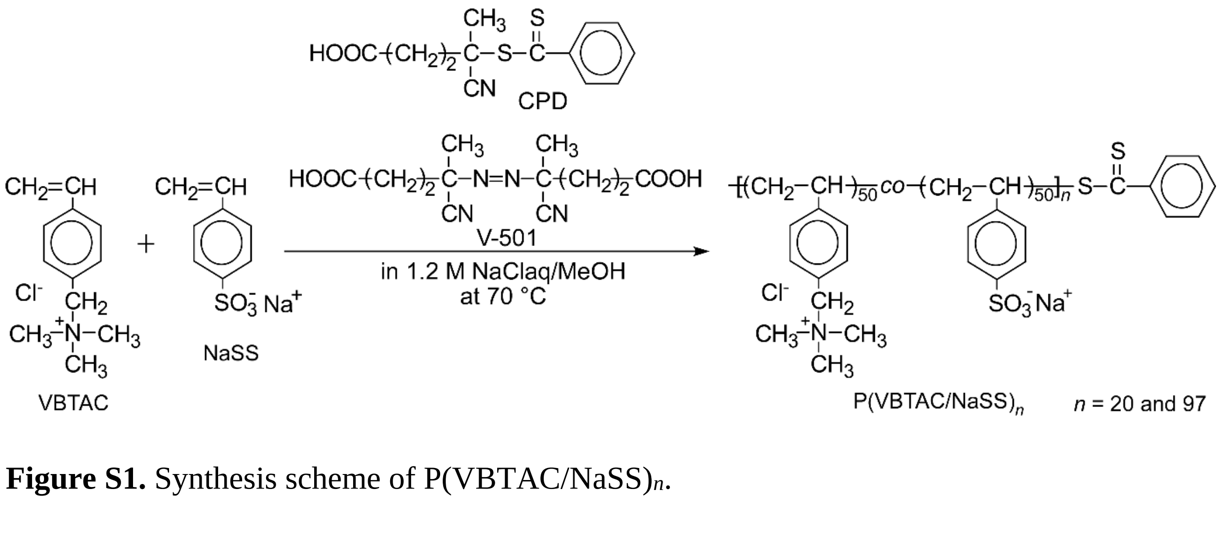

Figure S1. Synthesis scheme of P(VBTAC/NaSS)$_n$.

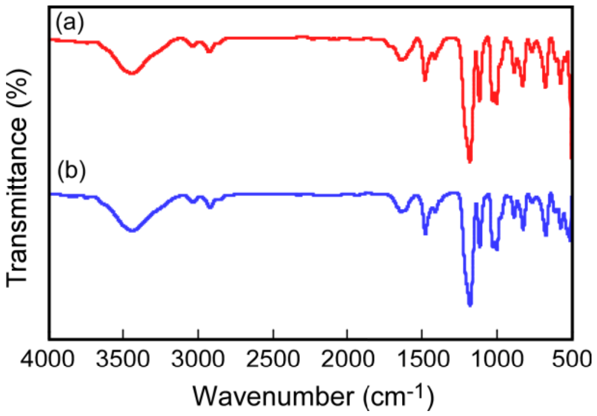

Figure S2. ATR-IR spectra for (a) P(VBTAC/NaSS)$_{20}$ and (b) P(VBTAC/NaSS)$_{97}$.

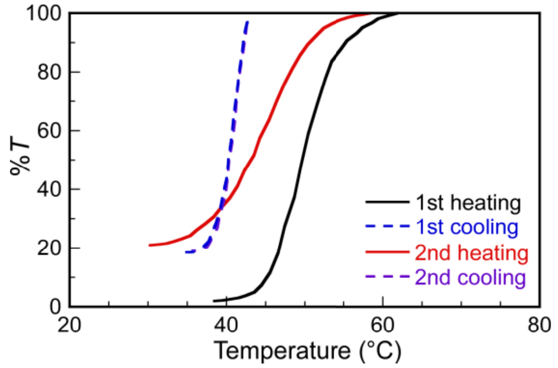

Figure S3. Percent transmittance (%*T*) at 700 nm for 0.1 M NaCl aqueous P(VBTAC/NaSS)$_{20}$ at C_p = 1.0 g/L as a function of temperature upon heating and cooling.

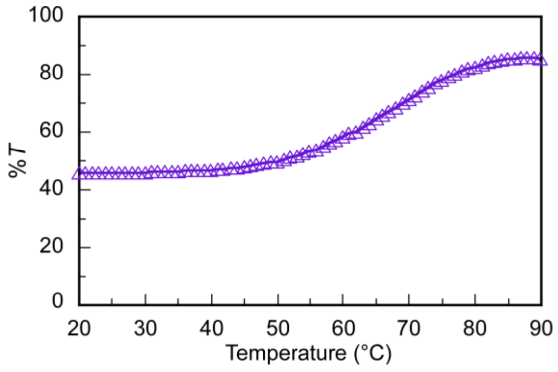

Figure S4. Percent transmittance (%*T*) at 700 nm for P(VBTAC/NaSS)$_{97}$ at C_p = 2.0 g/L in pure water as a function of temperature upon cooling.

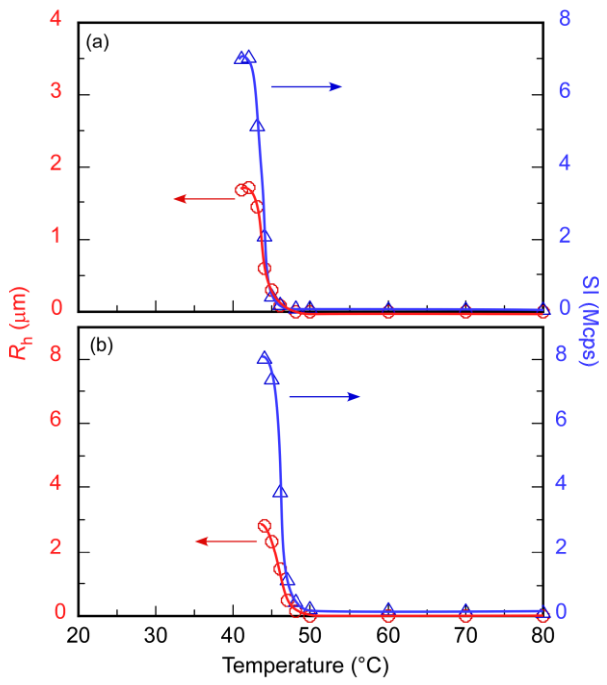

Figure S5. Hydrodynamic radii (R_h, ○) and light scattering intensities (SI, △) for (a) P(VBTAC/NaSS)$_{20}$ at [NaCl] = 0.1 M and (b) P(VBTAC/NaSS)$_{97}$ at [NaCl] = 1.0 M with C_p = 2.0 g/L as a function of temperature.

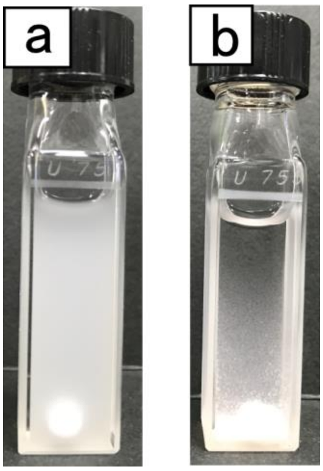

Figure S6. (a) Dispersion of P(VBTAC/NaSS)$_{97}$ just below the UCST and (b) flocculation of the polymer below the UCST upon standing for 2 h.

Table S1. Maximum fluorescence wavelength (λ_{max}) of PNA in the absence and presence of P(VBTAC/NaSS)$_n$ in aqueous solutions at 20 and 70 ℃

Sample	without polymer[a]		P(VBTAC/NaSS)$_{20}$[b]		P(VBTAC/NaSS)$_{97}$[c]	
Temperature (℃)	20	70	20	70	20	70
λ_{max} (nm)	463	452	412	417	417	423

[a]In pure water. [b]At C_p = 2.0 g/L in 0.1 M NaCl solutions. [c]At C_p = 2.0 g/L in 1.0 M NaCl aqueous solutions.

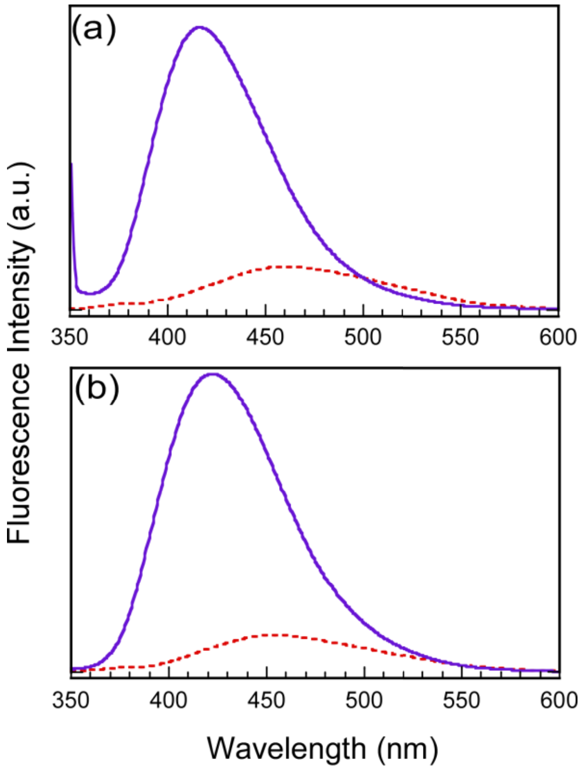

Figure S7. Typical examples of fluorescence emission spectra for PNA in the absence (----) and presence of P(VBTAC/NaSS)$_{97}$ (——) at C_p = 2 g/L in 1.0 M NaCl aqueous solutions at (a) 20 and (b) 70 ℃.

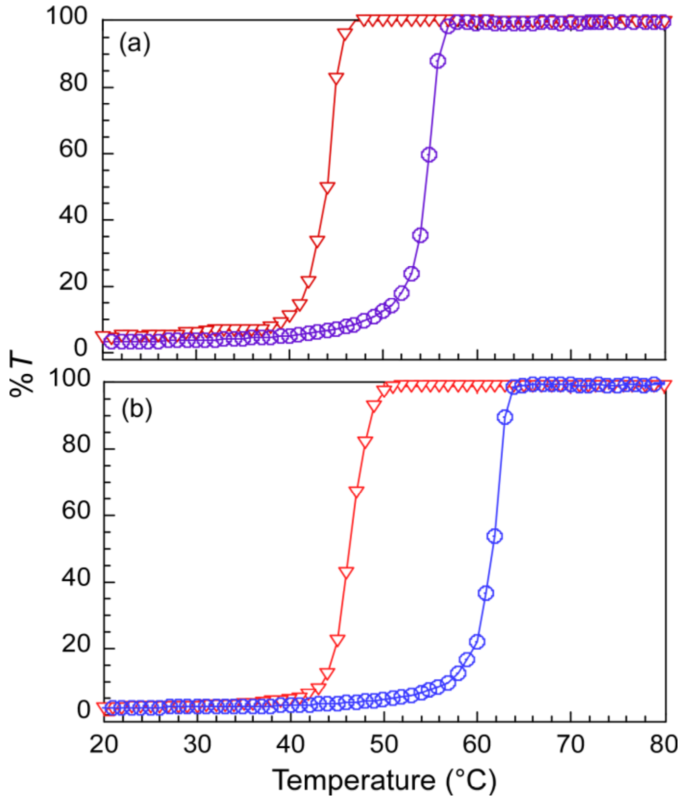

Figure S8. Percent transmittance (%*T*) at 700 nm for H_2O (▽) and D_2O (○) solutions of (a) $P(VBTAC/NaSS)_{20}$ at [NaCl] = 0.1 M and (b) $P(VBTAC/NaSS)_{97}$ at [NaCl] = 1.0 M with C_p = 2 g/L as a function of temperature.
